# Signaling Pathways in Proton and Non-proton ASIC1a Activation

**DOI:** 10.3389/fncel.2021.735414

**Published:** 2021-10-05

**Authors:** Libia Catalina Salinas Castellanos, Osvaldo Daniel Uchitel, Carina Weissmann

**Affiliations:** Instituto de Fisiología, Biología Molecular y Neurociencias (IFIBYNE—UBA CONICET), Facultad de Ciencias, Exactas y Naturales de la Universidad de Buenos Aires, Buenos Aires, Argentina

**Keywords:** ASIC1a, proton activation, non-proton activation, ERK, MitTx, pain

## Abstract

Acid-sensing ion channels (ASICs) regulate synaptic activities and play important roles in neurodegenerative diseases as well as pain conditions. Classically, ASICs are described as transiently activated by a reduced pH, followed by desensitization; the activation allows sodium influx, and in the case of ASIC1a-composed channels, also calcium to some degree. Several factors are emerging and extensively analyzed as modulators, activating, inhibiting, and potentiating specific channel subunits. However, the signaling pathways triggered by channel activation are only starting to be revealed.The channel has been recently shown to be activated through a mechanism other than proton-mediated. Indeed, the large extracellular loop of these channels opens the possibility that other non-proton ligands might exist. One such molecule discovered was a toxin present in the Texas coral snake venom. The finding was associated with the activation of the channel at neutral pH *via* the toxin and causing intense and unremitting pain.By using different pharmacological tools, we analyzed the downstream signaling pathway triggered either by the proton and non-proton activation for human, mouse, and rat ASIC1a-composed channels in *in vitro* models. We show that for all species analyzed, the non-protonic mode of activation determines the activation of the ERK signaling cascade at a higher level and duration compared to the proton mode.This study adds to the growing evidence of the important role ASIC1a channels play in different physiological and pathological conditions and also hints at a possible pathological mechanism for a sustained effect.

## Introduction

ASICs, also called proton-gated channels belong to the degenerin/epithelial Na^+^ channel gene family (Boscardin et al., 2016). Five genes encode at least seven ASIC subtypes in rodents and humans, and three subunits constitute a functional unit in either homotrimeric or heterotrimeric structures (Boscardin et al., 2016). These channels are primarily expressed in the nervous system (Zha, 2013) and linked to several physiological (Uchitel et al., 2019) and pathological conditions (Chu and Xiong, 2013), thus different pharmacological tools have been developed as potential therapeutic treatments.

ASICs are Na^+^-selective ion channels, and ASIC1a,—a key subunit in the central nervous system (Wang et al., 2016)—, show, in addition to its Na^+^ permeability, a small permeability to Ca^2+^ (Gründer and Chen, 2010). ASIC1a has been linked to neurodegenerative diseases (Friese et al., 2007; Wong et al., 2008; Sluka et al., 2009; Sun et al., 2011), ischemia (Xiong and Xu, 2012), and pain (Duan et al., 2007; Wemmie et al., 2013; Fan et al., 2018). The unique permeability to calcium compared with other subunits makes ASIC1a a candidate to play a prominent role in neuronal death (Hoagland et al., 2010).

Under experimental conditions, ASICs are activated only by rapid pH drops, and, particularly homomeric ASIC1a channels desensitize rapidly in the continuous presence of acidic pH (Chu and Xiong, 2013). This fact remains puzzling, as to whether a significant amount of ASIC1a current can be activated in pathological conditions, and as to whether the effect of its activation could be long-lasting (Chu and Xiong, 2013; Tikhonov et al., 2019; Alijevic et al., 2020); thus the functional significance of these channels remains to be determined. As pointed out by Zha (2013), although the canonical ligands for ASICs are protons, the massive extracellular domain of ASICs has led to the speculation that these receptors may also respond to other ligands (Zha, 2013) like MitTx purified from the venom of the Texas coral (Kweon and Suh, 2013). The toxin has been instrumental to document ASIC1a channels in an open state (Baconguis et al., 2014). MitTx elicits robust pain-related behavior in mice *via* activation of ASIC1 channels on capsaicin-sensitive nerve fibers (Bohlen et al., 2012).

Gautschi et al. (2017) on the unresolved question of the unphysiological pH values used to activate the channels and the transient nature of the proton evoked ASIC current, described another type of activation other than acid, as a “non-proton” mechanism (exemplified by MitTx), that activated a large sustained and non-desensitizing current at neutral pH and exceeding in magnitude the maximal current evoked by the proton mode (Gautschi et al., 2017).

Many studies have focused on the mechanism regulating the trafficking of the channel (Zeng et al., 2014; Boscardin et al., 2016; Wu et al., 2016), leading to changes in the amount of channel at the plasma membrane. The downstream signaling of ASIC channels, however, is only starting to be documented.

As an example, the activation of ERK *via* ASIC1a (downstream ASIC1a activation) has been analyzed in different pathological conditions (Chen et al., 2016; Sun et al., 2018; Zhu et al., 2020, 2021). In addition, this pathway has also been linked to inflammation (Yu et al., 2015). Conversely, the effect of MAP kinases on ASIC1a (upstream of ASIC1a activation) has also been analyzed in different scenarios (Duan et al., 2012; Aissouni et al., 2017; Peng and Kellenberger, 2021; Wei et al., 2021) especially in association to its effect on the insertion of channels, and thus increase of channels in the plasma membrane.

Work by Yu et al in striatal neurons established a critical link between ASIC1a activity and CaMKII-ERK signaling in the regulation of striatal synaptic remodeling (Yu et al., 2018). In addition, they showed up-regulation of the ERK pathway in HEK cells *via* acid activation of ASIC1a endogenous channels.

The ERK kinase belongs to the family of mitogen-activated protein kinases (MAPK) that operate within signaling cascades (Maik-Rachline et al., 2019). The activation of this pathway and the duration of the activation (Marshall, 1995; Kriegsheim et al., 2009) can lead to different biological responses that can determine the fate of a cell. In addition, the activation of the pathway has been implicated in pain research. Activation of the kinase *via* phosphorylation in the dorsal root ganglia has been linked to different models of pain in animals (Cruz and Cruz, 2007; Maruta et al., 2019). Different levels of activation of ERK were distinguished in response to acute noxious stimulation or chronic noxious stimulation by Cruz and Cruz (2007), with more intense levels of ERK phosphorylation and longer duration in animals with chronic inflammation of the hind paw or joint. In the study, spinal ERK activation was upregulated and became persistent (Cruz and Cruz, 2007).

In this study, we aim to address aspects of the downstream effects triggered by non-proton activation of ASIC1a channels.

## Materials and Methods

### Cellular and Molecular Biology

Human embryonic kidney 293 (HEK) cells [passage 18–26, American Type Culture Collection (ATCC) number CRL-1573] were maintained by serial passages. Primary striatal cultures were prepared from mice of the C57BL/6 genetic background as control and ASIC1a−/− mice (generated using mice of the C57BL/6 genetic background) were provided by the laboratory of Dr. John A. Wemmie (University of Iowa, Iowa City, IA) as used before (González-Inchauspe et al., 2017) and prepared according to the protocol used in Sodero et al. (2011). All experiments involving mice were performed following national guidelines for the humane treatment of laboratory animals from the University of Buenos Aires (CICUAL Protocol #112), which are comparable to those of the USA National Institutes of Health. For biochemical analysis, six or 12-plates were coated with 0.1 mg/mL of poly-L-lysine (PLL, Sigma, P2636), and dissociated neuronal and HEK cells were plated at a density of 2.2 × 10^5^ or 1.4 × 10^5^ cells respectively. HEK cells were grown in Dulbecco’s Modified Eagle’s Medium containing 4 mM L-glutamine, 4.5 g/L glucose, and 110 ml/L sodium pyruvate and supplemented with 10% Fetal Calf Serum (NatoCor). Transfection of the cells was performed with the calcium phosphate method as described previously (Weissmann et al., 2013). The eGFP-ASIC1a encoding plasmid used was a gift of Dr. Stefan Gründer. Transfected cells were used 2 days after transfection. Neurons were grown in Neurobasal medium™ (Thermo Fisher) with B27 supplement (Thermo Fisher) and used after 7–8 days *in vitro*. HEK and neurons were both kept at 37°C and under 5% CO_2_. For microscopy experiments, cells were plated on glass coverslips (12 mm rounded Carolina^®^ Assistant-Brand Cover), coated with 1 mg/ml of PLL (Sigma, P2636). All materials were purchased from Sigma unless stated otherwise.

### Drugs and Treatments

Incubation of cells: ASIC inhibitors were used at the following concentrations before incubation with other reagents: Pctx-1 (Alomone, STP-200), 20 nM, 30 min before; as previously used in Salinas et al. (2020). MitTx (Alomone, M-100) was used at a concentration of 20 nM for 2, 10 min according to Alomone Labs and (Bohlen et al., 2012). Solutions used for the different incubations were prepared as follows: for incubation of cells with the different reagents and controls were: solution at pH 7.3, containing the following (in mM): NaCl 128, KCl 2.5, CaCl_2_ 2, MgCl_2_ 1, glucose 15, sucrose 15, HEPES 5, MES 5 adjusted to pH 7.4; and for treatments to activate through the proton mechanism solution were adjusted to pH 6 with HCl (“pH6”).

### Western Blotting (WB)

Western blots were performed according to standard procedures. In brief, cells were resuspended in a 1% SDS HEPES pH 7.4 lysis buffer containing a protease inhibitor cocktail (Roche, cOmplete™); in the case of lysates used for detection of phosphorylated ERK, the buffer included 50 mM sodium fluoride, 2 mM sodium orthovanadate. Proteins were resolved by 4–10% polyacrylamide gels and transferred onto Immobilon^®^-FL PVDF membranes. Non-specific binding was blocked by 1% non-fat powdered milk in TBS containing 0.2% Tween-20 for 60 min at RT. Membranes were incubated overnight at 4°C with primary antibodies in 1% BSA in TBS, followed by the addition of secondary antibodies in 1% non-fat powdered milk in TBS.

The following primary antibodies were used: rabbit polyclonal anti ASIC1 (Alomone ASC-014, 1:1,000); mouse monoclonal anti-tubulin (DM1a; Cell signaling #3873, 1:5,000); rabbit polyclonal anti total ERK (Santa Cruz, C9, 1:500); rabbit polyclonal anti phosphoERK (Cell Signaling, SC-7383, 1:500); pCaMKII (Phosphosolutions, p1005-286). Initially, each antibody was detected in full membranes to verify that only the expected MW bands were present and the optimal dilution was decided upon. Accordingly, membranes were cut using MW standards as a guide to detect different proteins in the same membrane. No membrane stripping protocols were performed, thus bands of the same MW were obtained from the same samples run on different membranes. Reactive bands were detected by the LI-COR Odyssey system, using secondary antibodies: 926-68073 IRDye 680RD Donkey anti-Rabbit IgG or 926-32212 IRDye 800CW Donkey anti-Mouse.

Images were taken using the LI-COR Odyssey system and quantified with ImageJ software (NIH, USA).

### Detection of Proteins by Immunofluorescence (IF)

Cells grown on PLL-coated glass coverslips were fixated with 4% p- formaldehyde in PBS, permeabilized with 0.1% Triton x-100 (10 min), and treated with blocking solution (1% BSA, 0.01% Triton x-100 in PBS) for an hour at RT. Coverslips were incubated then with the primary antibody for overnight in blocking buffer, washed in PBS, and incubated with the secondary antibody for 60 min in blocking buffer. After a final wash in PBS, coverslips, were placed onto a slide and covered with a mounting medium. The antibodies used were rabbit polyclonal antibody against mouse monoclonal anti-tubulin (DM1a; Cell Signaling, #3873, 1:2,000); rabbit polyclonal anti-phospho ERK (Phosphosolutions, p160–202, 1:100). Images were taken using an Olympus FV300/BX61 microscope with a 60× (1.4 NA) oil-immersion objective. Alexa-647 and Alexa-488-conjugated secondary antibodies (ThermoFisher) were used.

## Results

### Activation of pERK Through ASIC1a *via* Non-proton Mechanisms

The effect of MitTx on the activation of ERK on mouse striatum cells was studied since the toxin can activate ASIC1a channels at neutral pH and for a longer duration (Bohlen et al., 2012). The effect was studied at the mouse striatum neurons as these cells are enriched in ASIC1 channels composed predominantly of ASIC1a subunits constituting homomeric channels (Jiang et al., 2009). Furthermore, Yu et al. (2018) showed at the striatum that a decrease in pH triggered the activation of the CaMKII signaling pathway leading to the activation of ERK kinases.

We decided to analyze whether the downstream effects of MitTx activation would lead to the same signaling pathways as those triggered by proton activation.

**Figure 1:**
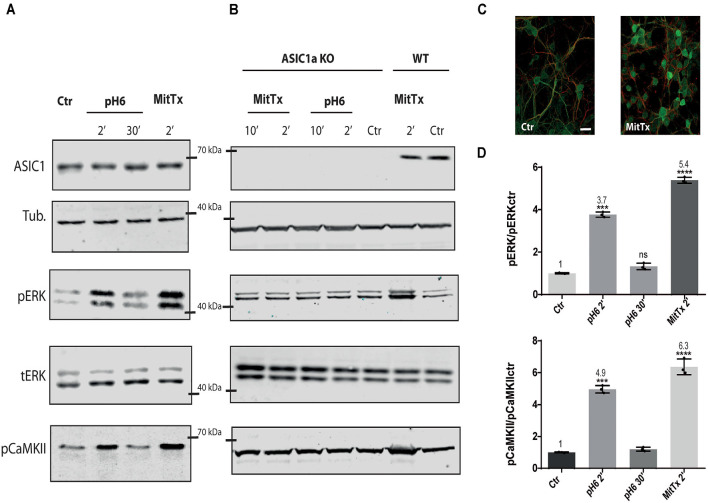
Non-proton activation of ASIC1a in striatal neurons. Representative membranes of lysates from 7 DIV wild type **(A)**, and ASIC1a knockout **(B)** C57 mice striatal neuronal cultures were incubated with MitTx and compared to treatment with pH6 solutions for the time indicated (2, 10, or 30 min). The detection was performed with anti ASIC1, tubulin (Tub), phospho ERK (pERK), total ERK (tERK), or phospho CaMKII (pCaMKII) antibodies, and Licor secondary antibodies. **(C)** Examples of images of striatal cultures used and treated with MitTx and stained with tubulin (red) and phosphoERK (green) antibodies and secondary Alexa fluor antibodies, 60× objective used. Scale bar 10 μm. **(D)** Result of the bands detected in **(A)** for pERK/ERKt levels relative to control samples showing an increase in both, pH6 or MitTx treatments and the same pattern of increase for pCaMKII. Notice the lack of effect in ASIC1a knock-out derived cultures. **(A)** Notice that plots are the result of the signal intensity of the bands detected for each antibody, and tERK and tubulin are used as loading controls between loaded samples. Data are presented as the mean ± SEM ANOVA and Dunnet *post hoc* test for treatments against the control were performed, mean values above bars; *n* = 3 membranes, ****p < 0.0001; ***p 0.0001–0.001; ns: no significant differences. Mean values expressed relative to control (**Ctr**) levels ± SEM are as follows: for pERK/tERK: **pH6 2’** 3.77 ± 0.07; **pH6 30’** 1.32 ± 0.09; **MitTx 2’** 5.39 ± 0.08. For pCaMKII/Tub:** pH6 2’** 4.97 ± 0.14; **pH6 30’** 1.20 ± 0.07; **MitTx 2’** 6.37 ± 0.28.

For this purpose, mouse striatal cultures were treated with MitTx and compared to cultures treated with acidic solutions. As shown in Figures 1A–D, MitTx-treated cultures evidence an increase in phospho ERK levels following the same pattern as phospho CaMKII activation which is much stronger than that shown for pH6-treated cultures at 2 min (as documented by Yu et al., 2018) or 30 min. The signal ratio of pERK/tERK for pH6 2’ treated cells is four times greater than control cells (*pH6 2’* 3.77 ± 0.07), and *MitTx for 2’* leads to more than a 5-fold increase (*MitTx 2’* 5.39 ± 0.08). This effect is not present in striatal cultures obtained from ASIC1a knock-out cultures and treated either with pH6 solutions or MitTx (Figure 1B).

### Proton and Non-proton Activation of ASIC1a Human Subunits

**Figure 2:**
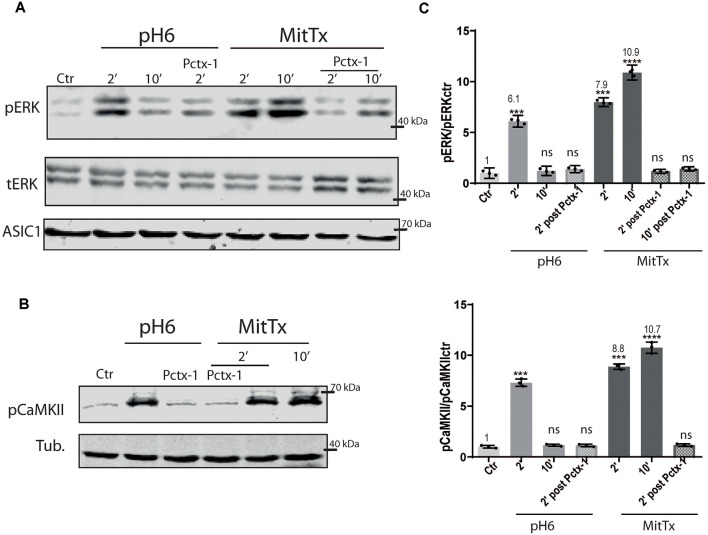
Proton and non-proton activation of ASIC1a in HEK cells. **(A)** Representative membranes of lysates of HEK cells treated with pH6 or MitTx for 2 or 10 min or preincubated with Pctx-1 compared to untreated cells (control, Ctr) and detected with phosphoERK (pERK), total ERK (tERK), and ASIC1 antibodies. **(B)** Representative membrane of the same lysates to detect pCaMKII levels. **(C)** Plots showing detected levels of pERK (top panel) or pCaMKII (lower panel). Notice that the increase in kinase levels goes further at a later time point (2 vs. 10 min) in MitTx treated cultures compared to pH6 treated ones that show an increase at 2 min followed by a reversal to control levels consistent with the proton-activated desensitizing mechanism. **(A)** Notice that plots are the result of the signal intensity of the bands—with tERK and tubulin used as loading controls between loaded samples—and expressed relative to control samples. Data are presented as the mean ± SEM ANOVA and Dunnet *post hoc* test for treatments against the control were performed, mean values above bars; *n* = 3 membranes, ****p < 0.0001; ***p 0.0001–0.001; ns: no significant differences. Mean values expressed relative to control (**Ctr**) levels ± SEM are as follows: for pERK/tERK: **pH6 2’** 6.10 ± 0.13; **pH6 10’** 1.23 ± 0.11; **pH6 2’** **Pctx** 1.39 ± 0.08; **MitTx 2’** 7.98 ± 0.10; **Mittx10**’ 10.90 ± 0.17; **MitTx 2’ Pctx** 1.18 ± 0.05**; MitTx 10’ Pctx** 1.42 ± 0.05. For pCaMKII/Tub: **pH6 2’** 7.31 ± 0.21; **pH6 10’** 1.16 ± 0.06; **pH6 2’ Pctx**1.14±0.07; **Mittx 2’** 8.88 ± 0.15; **Mittx 10’** 10.76 ± 0.32; **Mittx 2’ Pctx** 1.17 ± 0.07.

The effect of the non-proton activation of ASIC1a channels was analyzed further with MitTx on HEK cells that endogenously express ASIC1a subunits (Gunthorpe et al., 2001). Human, rat, and mice ASIC1a channels show differences, as shown for instance by a different degree of glycosylation that leads to different surface channel levels (Kadurin et al., 2008) and levels of activation (Xu et al., 2018). Therefore, we also tested the mechanism on this subunit. The activation of the pERK pathway has also been shown through the treatment of HEK cultures with pH6 solutions (Yu et al., 2018). Figure 2 shows the effects of either pH6 or MitTx treatments of cultures for different durations and also after incubation of cultures with Psalmotoxin (Pctx-1) a toxin that stabilizes the desensitized state of the channel constituted by ASIC1a subunits (Chen et al., 2005).

The degree of ERK phosphorylation is not only greater through the non-proton mechanism [compare *pH6 2’* 6.10 ± 0.13 (6-fold increase) vs. *MitTx 2’* 7.98 ± 0.10, 8-fold increase] but also, these levels increase in time compared to the transient activation of ERK *via* the proton mechanism (*MitTx 10’* 10.90 ± 0.17, 10-fold increase to control levels). In both cases, Pctx-1 can inhibit the activation of ERK to control levels. Phosphorylated ERK was detected even after 30 min incubation with MitTx (not shown).

### Effect of Proton and Non-proton Activation and Different Levels of ASIC Channels

Different pathological conditions show an increase in ASIC1a levels (Duan et al., 2007). To model this situation and analyze the signaling pathway triggered by the non-proton activation of the channel, we used HEK cells transfected with different levels of ASIC1a channels using a plasmid encoding for the rat ASIC1a subunit fused to eGFP (eASIC), thus distinctively detected in WB *via* the different molecular weights (due to the eGFP tag added), as used before (Salinas et al., 2020).

**Figure 3:**
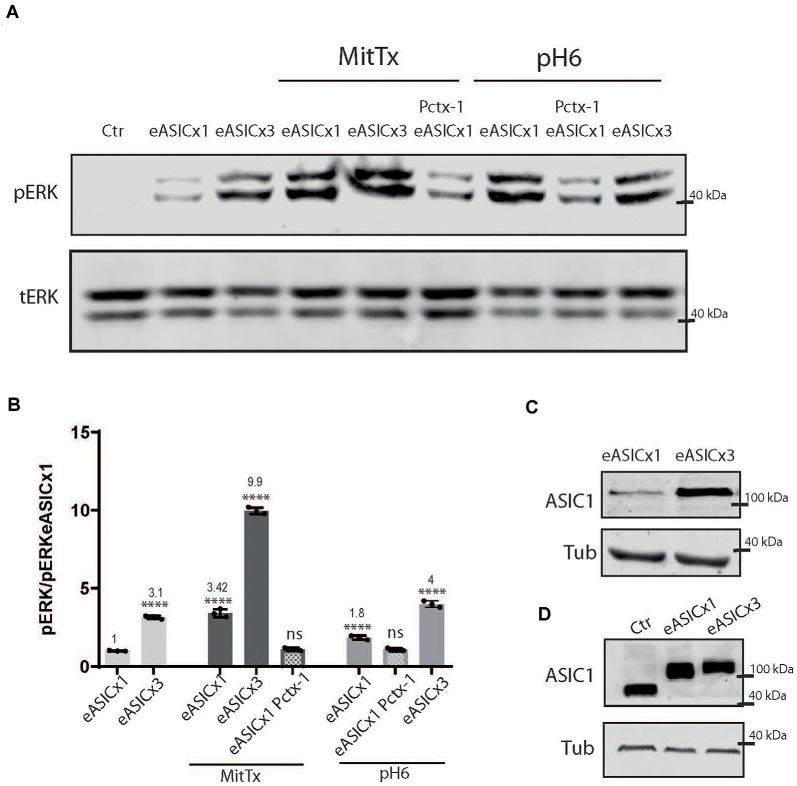
Proton and non-proton activation of overexpressed ASIC1a channels.** (A)** Representative membranes of lysates of cells control (ctr) or transfected with eGFP-ASIC1a (eASIC) at two levels (1x or x3) to obtained different levels of expression of the protein (“eASICx1 or eASICx3”), and treated with pH6 or MitTx with or without pre-incubation of Pctx-1 or untreated. **(B)** Plots showing the increase in pERK and pCaMKII levels calculated from membranes as that shown in **(A)**, consistent with the increase in eASIC expressed. Notice the level of increase achievable *via* MitTx incubation at the highest overexpressed level of eASIC, higher than that obtained *via* pH6. **(C)** Representative membrane showing the different levels of eASIC in cells overexpressing the channel (1x or 3x), detected with an ASIC1 antibody. **(D)** Comparison between the different ASIC1 proteins expressed (the endogenous human ASIC1a; of approx. 67 kDa) and the overexpressed eASIC (approx. 110 kDa, and expressed at different levels; x1 or x3). **(A)** Notice that plots are the result of the signal intensity of the band detected,—tERK and tubulin are used as loading controls between loaded samples—and expressed relative to eASICx1 levels. Data are presented as the mean ± SEM ANOVA and Dunnet *post hoc* test for treatments and conditions were performed, mean values above bars; *n* = 3 membranes, ****p < 0.0001; ns: no significant differences. Mean values expressed relative to **eASICx1** levels ± SEM are as follows: **eASICx3** 3.16 ± 0.06; **eASIC MitTx** 3.42 ± 0.15; **eASICx3 MitTx** 9.96 ± 0.12; **eASIC MitTx Pctx** 1.10 ± 0.05; **eASIC pH6 2’** 1.85 ± 0.08; **eASIC pH6 2’ Pctx** 1.08 ± 0.05; **eASICx3 pH6 2’** 4.00 ± 0.12.

As depicted in Figure 3, rat ASIC1a is also activated to a greater level when incubated with MitTx instead of pH6 (*MitTx* 3.42 ± 0.15 vs. *pH6* 1.85 ± 0.08). When HEK cells overexpress the channel (Figures 3B,C), the activation of the ERK pathway is increased at basal levels (compare **Ctr and eASICx1** bands, Figure 3A; and **eASICx3** 3.16 ± 0.06, normalized to eASICx1 levels), and is activated further *via* the proton or non-proton signaling mechanism. But as the increase becomes greater (compare transfection of plasmids to different levels, either “1x” or “3x”), the non-proton mechanism is still able to activate the channel to greater levels (**eASICx3 MitTx** 9.96 ± 0.12), whereas the proton activation is no longer able to reflect this change (Figures 3A,B). Interestingly, the pERK/tERK ratio of eASICx1-MitTx/eASICx1 and eASICx3-MitTx/eASICx3 remains about the same (3-fold increase).

## Discussion

In this study, we analyzed the signaling pathway triggered by the activation of ASIC1a channels through a non-proton mechanism.

We show that this mechanism determines the activation of the CaMKII-ERK pathway for a longer period than that resulting from proton activation. This mechanism was conserved for the different ASIC1a subunits analyzed (mouse, in Figure 1, human in Figure 2, and rat in Figure 3) in the different *in vitro* models. Furthermore, the mechanism could even reach higher levels if ASIC1a subunits were expressed at higher levels (Figure 3). The fact that mouse striatal KO cultures showed no evidence for this mechanism reinforces the argument that these mechanisms analyzed act *via* ASIC1a and no other pH-sensitive receptor.

The pathway (downstream ASIC1a activation) has been shown as signaling for different events relevant in physiological as well as pathological conditions (Kriegsheim et al., 2009). The mechanism, which is dependent on a stimulus that leads to the three-tier activation cascade with sequential kinase activation, has also been shown to crosstalk with the CaMKII pathway in many cells (Illario et al., 2003; Salzano et al., 2012). Accordingly, for some stimuli and cell models, CaMKII is necessary for ERK activation, and the activation of both has been shown for striatal cells through the proton-mediated activation of ASIC1a channels (Yu et al., 2018). Nevertheless, whether the mechanism requires the conducting channel is a matter of debate. We showed that the presence of Pctx-1 prevents this mechanism and that CaMKII is activated, but whether the mechanism could rely on a conduction-independent pathway cannot be ruled out. As an example, ASIC1a phosphorylation by RIP1 leading to necroptosis pathways does not rely on conducting channels (Wang et al., 2015, 2020). Future experiments will reveal more details on the mechanism.

A comparative analysis of both mechanisms analyzed in this work shows that the proton mechanism leads to transient activation of ERK which can no longer be detected after 5 min. Increases or a decrease in pERK levels were detected in previous work *via* ASIC1a. Amiloride significantly decreased (an approximately half-fold) the levels of CaMKKß and ERK phosphorylation in a cell line of hepatic fibroblasts stimulated by high glucose and PDGF (Wang et al., 2019). Zhu et al. (2020) showed a contribution of ASIC1a to increased ERK phosphorylation in the mechanism of liver fibrosis, as Pctx-1 treatment decreased the approximate 2-fold increase (without treatment) to a 1.5-fold increase. The same can be observed in ERK phosphorylated levels as the bands show a greater intensity for ERK-mediated NF-κB activation through ASIC1 in response to acidosis (although not quantified; Chen et al., 2016). In striatal and HEK cells, Yu et al. (2018) showed an increase in phosphorylation levels through pH 6 incubation reaching approximately 230% for ERK1 and 250% for ERK2 higher levels than control cells. In this work, we detected an increase in total phosphorylated ERK levels *via* the proton mechanism. In contrast, the non-proton activation of ASIC1a channels leads to the phosphorylation of ERK to a greater extent and for a longer period, as no desensitization is present (still active at 30 min). Thus, the activation of the channel in a non-proton mechanism (as in a Texas coral snake bite) would trigger sustained phosphorylation of ERK that could lead to further signaling. Additionally, we noted that the increased phosphorylation of ERK (measured as the signal detected in pERK to tERK levels) reached the same level whether channels were expressed at higher levels as if the signaling could be the result of a fraction of occupied receptor mechanism (Andrews et al., 2016) that should be analyzed in the future.

The kinetics of ERK phosphorylation has been the subject of various studies (Kriegsheim et al., 2009; Ahmed et al., 2014; Shindo et al., 2016; Maik-Rachline et al., 2019). These studies revealed different aspects of the complexity in the regulation of ERK signaling, providing mathematical models accounting for different levels of regulation. Among these, negative feedback on ERK activation through upregulation of phosphatases that dephosphorylate ERK, as well as depletion of the stimulus (either through internalization or removal from the extracellular medium; Cirit et al., 2010) were shown to play a role in the transient shape of the signal. Additionally, ERK translocation to the nucleus and binding to cytosolic and nuclear substrates and dephosphorylation was also shown to play a main role in the kinetics of ERK signaling (Ahmed et al., 2014). Indeed, the translocation of ERK was later interpreted as a key to transforming a graded response (stimulus activating ERK) into a switch (ERK translocated to the nucleus) that can determine the fate of a cell (Shindo et al., 2016). Thus, ERK phosphorylated transiently (up to 5 min) or in a sustained manner determines a different biological response.

Nuclear ERK can determine the stabilization of immediate early gene products that can trigger further effects as observed by c-fos-mediated signaling when ERK is activated in a sustained manner (Murphy et al., 2002).

Our studies show that the proton-mediated activation of ASIC1a channels acts transiently activating ERK, the channel is desensitized and can no longer trigger the activation mechanism. This could be comparable to depletion of the stimulus.

The ERK pathway has been described as a network functioning as a potential switch, oscillator, or memory (Shindo et al., 2016). All these mechanisms concerning pain could lead to acute or persistent effects. Whether endogenous ligands for ASIC1a exist, however, remains to be determined. Nevertheless, the possibility that this pathway might explain aspects of pain warrants further analysis for potential therapies.

## Data Availability Statement

The original contributions presented in the study are included in the article, further inquiries can be directed to the corresponding author.

## Ethics Statement

The animal study was reviewed and approved by CICUAL, University of Buenos Aires, Argentina.

## Author Contributions

ODU and CW contributed to the conception and design of the study. LCSC performed experiments and the statistical analysis. ODU and CW wrote the manuscript. All authors contributed to the article and approved the submitted version.

## Conflict of Interest

ODU, coauthor to this manuscript is also editor of this special topic. The remaining authors declare that the research was conducted in the absence of any commercial or financial relationships that could be construed as a potential conflict of interest.

## Publisher’s Note

All claims expressed in this article are solely those of the authors and do not necessarily represent those of their affiliated organizations, or those of the publisher, the editors and the reviewers. Any product that may be evaluated in this article, or claim that may be made by its manufacturer, is not guaranteed or endorsed by the publisher.

## Abbreviations

ASIC: Acid Sensing Ion Chanel
eASIC: eGFP-ASIC1a
eASICx1 or eASICx3: eGFP-ASIC1a expressed at a single or triple-level
ERK: extracellular signal-regulated kinase
CaMKII: Calcium/Calmodulin kinase II.
